# Philadelphia chromosome positive AML arising from JAK2-positive myelofibrosis

**DOI:** 10.1186/s40364-018-0147-6

**Published:** 2018-11-21

**Authors:** Marte Karen Brattås, Kyrre Lilleeng, Randi Hovland, Ingvild Jenssen Lægreid, Marta Vorland, Friedemann Leh, Øystein Bruserud, Bjørn Tore Gjertsen, Håkon Reikvam

**Affiliations:** 1 Department of Medicine, Haraldsplass Deaconess Hospital, Bergen, Norway; 2 Department of Medical Genetics, Haukeland University Hospital, Bergen, Norway; 3 Department of Laboratory Medicine, University Hospital of Northern Norway, Tromsø, Norway; 4 Department of Medical Biochemistry, Haukeland University Hospital, Bergen, Norway; 5 Department of Pathology, Haukeland University Hospital, Bergen, Norway; 6 Department of Clinical Science, University of Bergen, Bergen, Norway; 7 Department of Medicine, Haukeland University Hospital, Bergen, Norway

**Keywords:** Primary myelofibrosis, AML, JAK2, Philadelphia chromosome, Clonal evolution

## Abstract

**Background:**

A feature of myeloproliferative neoplasia is transforming to more aggressive and malignant myeloid neoplasia, including acute myeloid leukemia. Different pathogenesis mechanisms participate in transformation, including transformation of existing potential preleukemic clones, since *JAK2*-mutant myeloproliferative neoplasms often transform to *JAK2* wild-type acute myeloid leukemia.

**Case presentation:**

Here, we present an 80 year old man with a *JAK2*-V617F mutant primary myelofibrosis. After 10 months the disease transform into a Philadelphia chromosome positive acute myeloid leukemia, detecting the cytogenetic aberration; t(9;22)(q34;q22) encoding the rare *BCR-ABL1* fusion gene; e6a2. The patient had treatment response to tyrosine kinases, illustrating the potential benefits of such approach in treating these patients subset.

**Conclusion:**

The case illustrates the potential of leukemic transformation to Philadelphia chromosome positive myeloid malignancies from potential existing preleukemic clones, and the awareness of such an evolution among patients with myeloproliferative neoplasms. Tyrosine kinases have potential effect also in patients presenting without chronic myeloid leukemia and with rare *BCR-ABL1* fusion transcripts, and should probably be a part of the treatment approach.

## Introduction

The Philadelphia chromosome (Ph) is a diagnostic feature for chronic myeloid leukemia (CML); Ph^+^ CML is identified by the genetic translocation t(9;22)(q34;q11.2) [1], that involve the fusion of the Abelson oncogene (*ABL1*) with the breakpoint cluster region (*BCR*) gene. The malignant transformation is hence caused by the acquisition of the fusion tyrosine kinase BCR-ABL1 in a hematopoietic stem cell, pivotal in transforming of the stem cell into a leukemic stem cell (LSC) that self-renews, proliferates, and differentiates to give rise to Ph^+^ acute myeloid leukemia (AML), acute lymphoblastic leukemia (ALL), or more frequently CML [2, 3]. The complete carcinogenesis of BCR-ABL1 is incompletely understood, since as many as 10% of healthy individuals may have this fusion transcript [4], presence of low level of *BCR-ABL1* transcripts seems to increase with increasing age [5], and in those individuals that develop BCR-ABL1 positive CML nearly half of the patients have additional mutations frequently found in myeloid disorders [6]. However, Ph^+^ AML developing after a previous Ph^−^ myeloproliferative neoplasia (MPN) or myelodysplastic syndrome (MDS) is rarely described. In this report we describe a patient with *JAK2V*617F positive primary myelofibrosis (PMF) that progressed to secondary Ph^+^ AML with gain of the rare *BCR-ABL1* fusion transcript e6a2.

## Case report

**Fig. 1:**
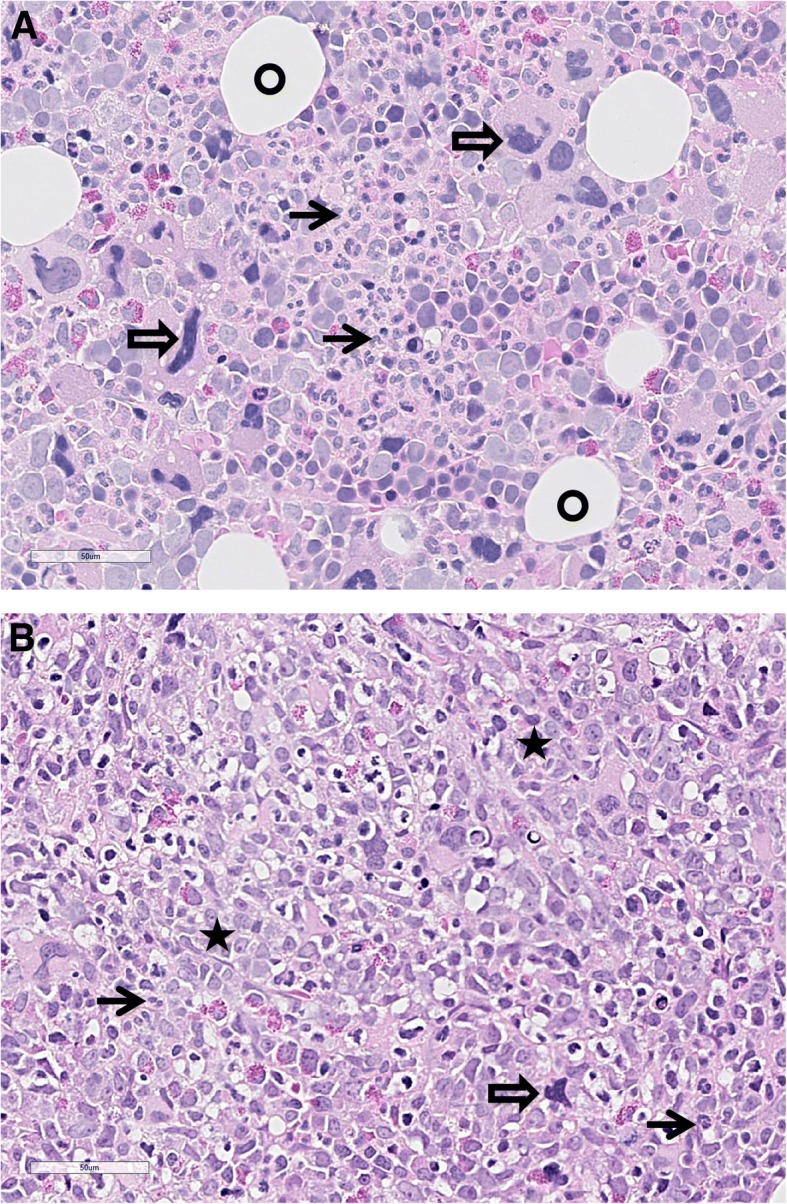
Histopathological examination in hematoxylin-eosin. **a**: BM at the initial diagnosis of PMF, showing hypercellularity with some fat cells left (circle), clusters of mature granulocytes (closed arrow), singular and groups of atypical megakaryocytes (open arrow). **b**: BM at follow up, showing maximal cellularity without any fat cells left, no organized hematopoiesis with only singular mature granulocytes, a few atypical megakaryocytes, and clusters of immature cells and blast cells (asterisk). (Pictures: Friedemann Leh, Department of Pathology, Haukeland University Hospital)

The patient was an 80 years old man whose previous medical record included diabetes mellitus type 2, atrial fibrillation, cerebrovascular disease, polymyalgia rheumatica and osteoporosis. His regular prescriptions included metformin, warfarin and prednisolone. He was admitted to hospital with a hematoma at his right thigh after a minor trauma. At clinical examination palpable splenomegaly at inspiration was detected. Standard peripheral blood tests revealed hemoglobin (Hgb) 10.3 g/dL (normal range 13.4–17.0), platelets > 2000 × 10^9^/L (150–450), white blood cell count (WBC) 23× 10^9^/L [4–11] and lactate dehydrogenase (LDH) 366 U/L (115–255). Microscopy of the peripheral blood smear revealed a leukoerythroblastic picture including nucleated erythrocytes and promyelocytes as well as myelocytes but no blasts. The bone marrow (BM) smear demonstrated increased cellularity with increased megakaryocytes and 4% myeloblasts; the BM biopsy confirmed this and showed in addition focal bundles of reticulin fibers and in addition proliferation of megakaryocytes with classic atypia, including small size and hypolobulation. There was reduced myelopoiesis, although without evidence of proliferation of immature cells (Fig. 1). Mutational analysis for *JAK2*V617F derived from peripheral blood mononuclear cell (PBMC) was positive with an allele burden of 0.6%, and a real time polymerase chain reaction (RT-qPCR) detecting the most common *BCR-ABL1* fusions; e13a2/e14a2/e1a2/e19a2, was negative. Hence, our patient fulfilled all WHO major criteria for a Ph^−^ myeloproliferative neoplasia; namely primary myelofibrosis (PMF); with (i) megakaryocytic proliferation and reticulin fibrosis, (ii) the presence of *JAK2* mutation and (iii) not fulfilling the criteria for other myeloid malignancies. In addition to all the five minor criteria were also fulfilled with (i) anemia, (ii) leukocytosis, (iii) palpable splenomegaly, (iv) increased LDH and (v) leukoerythroblastosis [7].

Cytostatic treatment with hydroxyurea was initiated at a dose of 2500 mg daily. The dose was reduced after some weeks due to severe headache. During the next 6 months the platelet count was reduced by hydroxyurea, although with difficulties in achieving satisfactory platelet counts without imposing neutropenia as a side effect. A shift of treatment to anagrelide (1 mg/day) was attempted, however had to be disrupted due to unacceptable side effects with headache, heart palpitations and back pain.

Seven months after the diagnosis of PMF the routine peripheral blood smear showed an increasing blast percentage and flow-cytometric analysis verified 22% immature cells. However, a BM biopsy demonstrated only 7% blasts. The diagnosis of PMF was therefore maintained and hydroxyurea continued.

Six weeks later the patient was admitted to hospital because of increasing anemia (Hgb 6.7 g/dL), leukocytosis (25.2 × 10^9^/L) and CRP 35 mg/L. Peripheral blood smear showed 43% myeloblasts, confirmed by flow cytometric analysis. BM biopsy demonstrated a hypercellular BM without organized hematopoiesis, absence of erythropoiesis and increased myelopoiesis with relatively few mature granulocytes, focal nodes of immature cells and blast cells and significantly increased amount of reticulin fibers (Fig. 1). The findings were consistent with transformation from PMF to AML.

**Fig. 2:**
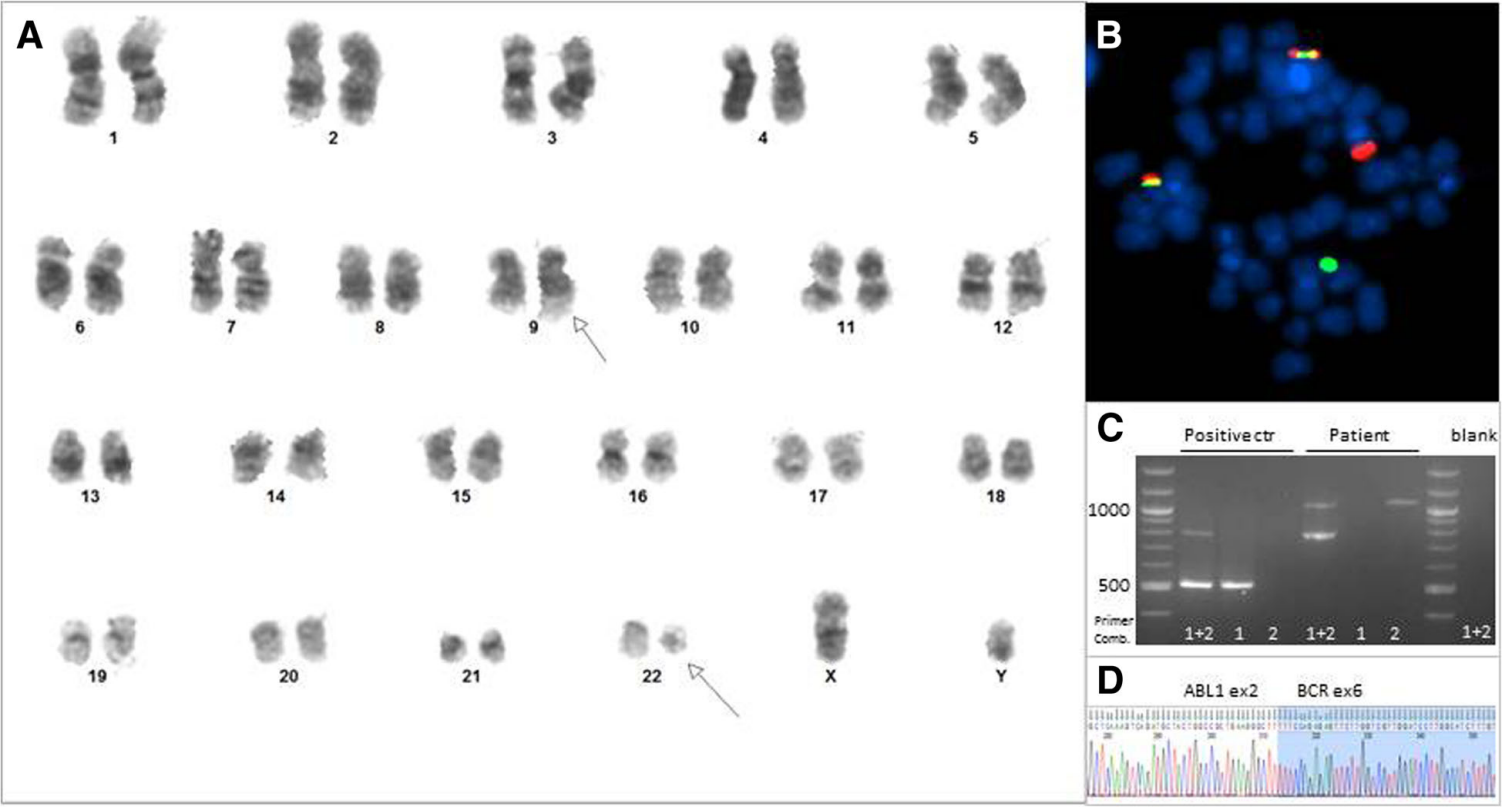
Chromosome analysis for detection of Philadelphia chromosome t(9; 22) in the patient. G-banding of diagnostic sample revealed a t(9;22)(q34;q11) (**a**) resulting in fusion of BCR and ABL verified by double fusion FISH from Vysis (**b**) and multiples RT-PCR (**c**). The multiplex (1 + 2) showed a 796 bp internal control (BCR) and 1051 bp BCR-ABL1 product generated by primer-combination BCR exon 1 and ABL1 exon 3 (2). The size gave suspicion of an e6a2 fusion verified by sanger sequencing using primer combination 2 (reverse sequence). (Picture: Randi Hovland, Haukeland University Hospital)

**Fig. 3:**
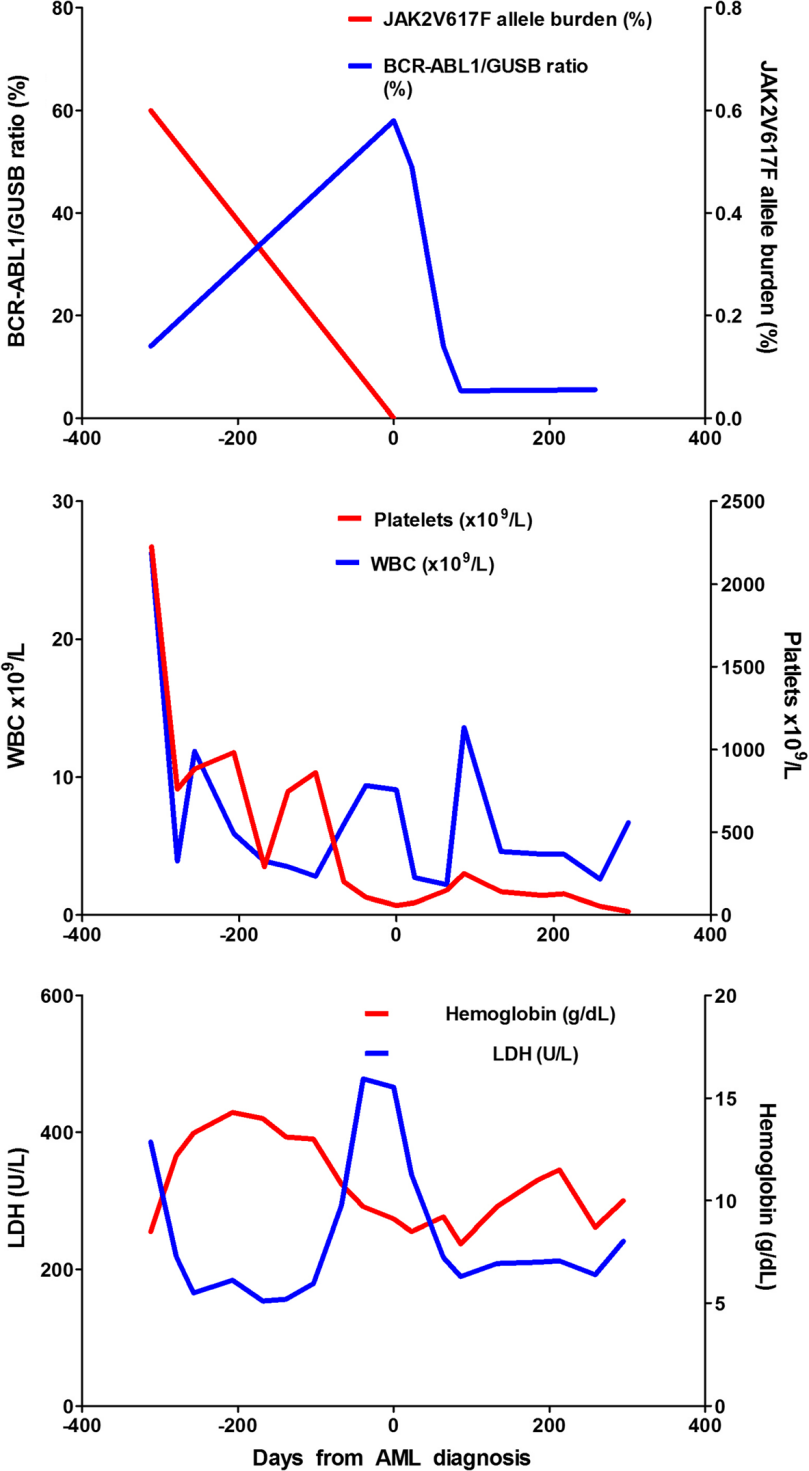
Development of genetically, hematological and biochemical values during the follow up of the patient. The diagnosis of AML is set to time point 0, and the negative values indicate days before the AML diagnosis, and the positive value days after the AML diagnosis

Surprisingly the cytogenetic analysis by conventional G-banding detected the Philadelphia chromosome with the translocation t(9;22)(q34;q22) in all ten metaphases analyzed. This was confirmed by *BCR-ABL1* fusion in 57% of the cells by fluorescent in situ hybridization (FISH) analysis, (Fig. 2) and to be the *BCR-ABL1* e6a2 transcript variant by sequencing of positive product from reverse transcriptase PCR. RT-qPCR confirmed the existence of an e6a2 *BCR-ABL1* transcript, with a *BCR-ABL1/GUSB* ratio of 69%. Retrospectively, the e6a2 transcript was also detected at the initial diagnosis of PMF, although only with a *BCR-ABL1/GUSB* ratio of 14% (Fig. 3). The *JAK2*V617F mutation could not be detected at the point of AML diagnosis.

We started treatment with dasatinib 100 mg once daily combined with hydroxyurea (500 mg/day) for the first 22 days and valproic acid (300 mg + 600 mg/day) for the first 20 days [8]. Peripheral blood smears after 11 and 19 days showed no myeloblasts. The *BCR-ABL1/GUSB* ratio fell from 52 to 7.6% after 3 months (Fig. 3). Because of increasing fatigability, the patient was referred to an echocardiography that showed a pericardial effusion of 1.8 cm at the level of the right atrium. This was regarded as an adverse effect of dasatinib [9], and serous effusions trigged by dasatinib is suggested to be predictive for therapy efficiency in CML [10, 11]. Due to risk of recurrent pericardial effusion combined with the general condition of the patient dasatinib therapy was discontinued after a treatment period of approximately 4 months.

Five days after dasatinib discontinuation, treatment with imatinib 400 mg daily was started, and a repeated echocardiography 2 weeks later demonstrated reduction in the pericardial effusion to 1.0 cm. At his last visit 2 weeks after initiating imatinib the patient reported that he had gradually improved. He experienced two episodes of diarrhea, but no other side effects of imatinib. Peripheral blood test showed Hgb 11.5 g/dL, WBC 5.3 × 10^9^/L, neutrophils 2.8 × 10^9^/L, and platelets 161 × 10^9^/L. He continued imatinib therapy for 6 months and had detectable although stable levels of *BCR-ABL1/GUSB* ratio measured by qPCR during this period (Fig. 3). Thereafter the patient developed increasing abdominal pain and diarrhea. A CT scan demonstrated a tumor in the pancreatic head, radiological consistent with adenocarcinoma. The patient was considered inoperable and unable to tolerate chemotherapy, and the tumor was not biopsied. He continued the imatininb treatment for an additional period of few weeks and died shortly thereafter.

## Discussion

Myeloproliferative neoplasms (MPNs) comprise a group of clonal stem cell disorders characterized by a high prevalence of mutations in one of the three genes *JAK2*, *CALR* or *MPL* [12], overproduction of mature blood cells, and variable rates of transformation to AML [13]. MPN comprise a wider spectrum of mutations, and the combination of mutations seems to predict progression rate to more aggressive myeloid neoplasms like AML [14]. Our patient was initially diagnosed with PMF, as he fulfilled all the major and minor diagnostic criteria as stated by the WHO guidelines [7]. He received cytoreductive treatment for lowering of the platelets count [15]. Accordingly, his platelet counts fell, and he had no sign of thrombotic disease.

The risk of transformation to more aggressive myeloid malignancies should always be kept in mind during follow up of for MPN patients. Older age by itself seems to be the main independent risk factor for transformation [16], although genotoxic therapy also seems to increase the risk [16]. The risk is higher for patients with PMF than for patients diagnosed with polycythemia vera (PV) and essential thrombocytosis (ET). Our patient developed increasing thrombocytopenia and raising WBC counts during follow up (Fig. 3), and a blood smear demonstrated an increasing blasts count. Development of AML was therefore suspected and verified by bone marrow examination showing a blast count > 20% [7] (Fig. 1). Hence, the patient fulfilled the criteria for secondary AML (s-AML). Surprisingly, the karyotyping demonstrated the presence of a Ph chromosome, confirmed by FISH analysis. Hence the myeloid malignancy was classified as Ph^+^. The Ph chromosome is a hallmark for CML, where > 97% of the patients present this translocation. However, it is believed that a low fraction (~ 1%) of AML patients also has this features [17]. Consequently, this is defined as an own entity with prognostic impact in the new recommendations from European Leukemia NET [18]. Although, the distinction between CML in blast phase and AML or bi-lineage acute leukemia (BI-AL) is not straightforward. Patients with Ph^+^ AML seem to have distinct morphological, clinical and genetical features distinguishing them from CML in blast phase [19, 20], while immunophenotypic features distinguish Ph^+^ AML from Ph + ALL and Ph + BI-AL.

In contrast to CML in blast phase, in which the disease mandatory harbors a *BCR-ABL1* fusion, AML transformation following a *JAK2*V617F-positive MPN commonly lacks the *JAK2*V617F mutation and thus presents with a *JAK2* wild-type leukemia [21, 22]. This was also the case in our patient, as the *JAK2*V617F mutation detected at diagnosis of PMF was no longer detected at the diagnosis of AML. Contrariwise, the *BCR-ABL1* e6a2 fusion gene detected at the diagnosis of AML was only detected retrospectively, and thus represents a minor cell subset at the time of PMF diagnosis. Taken together, these results indicate that the *BCR-ABL1* positive AML clone arose from a *JAK2* wild type cells and not from the clone giving rice to the initial PMF.

The *JAK2*V617F mutation allelic burden was low at the time of diagnosis, however low allelic mutation burden are not uncommon in PMF [23–25], in fact low *JAK2*V617F allele burden in PMF is associated with a more aggressive disease and poor overall survival [23, 24]. It has been postulated that an overriding *JAK2*V617F-negative clone conferring a more aggressive disease phenotype could be present [23–26], and for our patient we retrospectively detected this clone as being Ph^+^.

Our patient presented with the very rare *BCR-ABL1* transcript e6a2 that has been reported only for a few patients, and we have summarized these findings in Table 1. To the best of our knowledge this is the first report of this rare transcript arising in a previously *JAK2*V617F mutated patients. Clinically, CML cells with this variant e6a2 *BCR-ABL1* fusion transcript, often present in advanced stage with an aggressive disease, including presentation in blast phase and with AML. However, occasionally good responses to TKI have also been documented, a rare phenomenon reported in the case of Ph^+^ AML. For our patient, first dasatinib and later imatinib, resulted in considerable reduction of the *BCR-ABL1* transcript (Fig. 3). There is no evidence to support the preference for one TKI in Ph^+^ AML [18], although given the broader spectrum of kinase inhibition with dasatinib compared to other TKIs, some authors suggest dasatinib to be the TKI of choice [27].

**Table 1:** Clinical features of previously published patients with the e6a2 transcripts

Age/Sex	Disease	Transcripts	Treatment	Response to TKI	Clinical course/outcome	References
80/M	Secondary AML	e6a2	Dasatinib, Imatinib	Reduction in *BCR-ABL1* transcripts	Death from pancreatic tumor	Brattås et al., present
50/M	Chronic phase CML	e6a2	α interferon, Cytarabine	Not reported	Not reported	[29]
57/M	Chronic phase CML	e6a2	Imatinib, Dasatinib	Minimal cytogenetic response	Not reported	[30]
53/F	De novo AML	e6a2	Anthracycline based chemotherapy, Dasatinib, Imatinib	Complete molecular response	Not reported	[31]
76/M	Chronic phase CML	e6a2	Hydroxyurea, α interferon	Not reported	Death from cerebral ictus	[32]
65/M	Blast phase CML	e6a2	Hydroxyurea, Imatinib	Reduction of WBC after 30 days	Death from pneumonia	[33]
41/M	Chronic phase CML	e6a2	Hydroxyurea, Irradiation	Not reported	Death from sepsis 16 days after ASCT	[34]
55/F	De novo AML	e6a2	ASCT, Imatinib, Dasatinib, Nilotinib	Reduction of *BCR-ABL1* transcripts	Complete molecular remission	[35]
48/F	Blast crisis CML	e6a2	Imatinib	Reduction of *BCR-ABL1* transcripts	Complete molecular remission	[36]
67/M	Chronic phase CML	e6a2	Imatinib	Complete hematologic and cytogenetic response	Hematologic remission	[37]
37/M	Chronic phase CML	e6a1, e1a2	Imatinib	Partial molecular response	Disease stabilized on imatinib	[38]
48/M	Chronic phase CML	e6a2	Imatinib, Hydroxyurea, Dasatinib	Disease progression and resistance mutations	Death from blast crisis	[39]
42/M	Accelerated phase CML	e6a2	Imatinib, Dasatinib, ASCT	Persistent disease	Developed myeloid sarcoma	[40]
36/M	Chronic phase CML	e6a2, e1a2	Imatinib, Nilotinib, ASCT	Imatinib, Nilotinib	Progression to acute phase Complete cytogenetic remission	[41]
64/F	CMML	e6a2	Imatinib	Reduction of *BCR-ABL1* transcripts	Not reported	[42]
Not reported	CMML	e6a2	Induction chemotherapy, Dasatinib, Nilotinib	Disease progression despite reduction of Ph + clone	Death due to disease progression	[43]
Not reported	Blast crisis CML	e6a2	Dasatinib	Reduction of *BCR-ABL1* transcripts	Not reported	[44]
77/F	Accelerated phase CML	e6a2	Imatinib, Nilotinib	Complete hematological and cytogenetic response	Disease stabilized on nilotinib	[45]
53/F	De novo AML	e6a2	Imatinib, Dasatinib, ASCT	Complete hematological and cytogenetic response	Disease stabilized on dasatitinib	[46]
51/M	Accelerated phase CML	e6a2	Hydroxyurea, Imatinib, ASCT	Persistent disease	Not evidence of *BCR-ABL1* transcripts day + 30 after the second transplant	[47]
43/M	CML	e6a2	Imatinib	Complete hematological and cytogenetic response	Not reported	[48]

*Abbreviations*: *AML* acute myeloid leukemia, *ASCT* allogenic stem cell transplantation, *CML* chronic myeloid leukemia, *CMML* chronic myelomonocytic leukemia, *CMR* complete molecular response, *F* Female, *M* Male, *TKI* tyrosine kinase inhibitors, *WBC* white blood cells

Our patient developed a tumor in the pancreatic head. The tumor was not biopsied as the patient was considered unable to tolerate chemotherapy or surgery, and the diagnosis of pancreatic tumor was based on findings by CT scan, and considered radiological consistent with adenocarcinoma. Autopsy was not performed, so we can for sure not rule out a myeloid sarcoma as an uncommon manifestation of AML, although this seem unlikely. Interestingly, patient with MPNs probably have a modest, although significant, increased risk of secondary malignancies [28], as also detected in the present patient.

To the best of our knowledge this is the first report of a secondary Ph^+^ AML arising from previously *JAK2* mutated MPN. The very rare *BCR-ABL1* transcript e6a2 was detected in the transformed AML cells, and this transcript is associated with an aggressive phenotype. The AML arouse possibly from a more resistant but less robust primitive ancestral clone. The case illustrates the importance of new genetic evaluation also in s-AML since TKIs can be a treatment approach for such patients.

## Abbreviations

ABL1: Abelson
ALL: Acute lymphoblastic leukemia
AML: Acute myeloid leukemia
ASCT: Allogenic stem cell transplantation
BCR: Breakpoint cluster region
BI-ALL: Bi-lineage acute leukemia (BI-AL)
BM: Bone marrow
CRP: C-reactive protein
CML: Chronic myeloid leukemia
CMML: Chronic myelomonocytic leukemia
CMR: Complete molecular response
CT: Computed tomography
ET: Essential thrombocytosis
F: Female
FISH: Fluorescent in situ hybridization
Hgb: Hemoglobin
LDH: Lactate dehydrogenase
LSC: Leukemic stem cell
M: Male
MPN: Myeloproliferative neoplasia
PBMC: Peripheral blood mononuclear cell
PCR: Polymerase chain reaction
Ph: Philadelphia chromosome
PMF: Primary myelofibrosis
PV: Polycythemia vera
RT-qPCR: Quantitative real time polymerase chain reaction
s-AML: Secondary acute myeloid leukemia
TKI: Tyrosine kinase inhibitor
WBC: White blood cells
WHO: World Health Organization
